# Pregestational BMI and higher offspring’s risk of overweight/obesity in smoker and non-smoker mothers – CORRIGENDUM

**DOI:** 10.1017/S1368980022000775

**Published:** 2022-06

**Authors:** Nerea Martín-Calvo, Susana Santiago Neri, Gloria Segura, Cesar I Fernandez-Lazaro, María Barbería-Latasa, Miguel-Ángel Martínez-González

The authors regret the inclusion of an error in the above article. The affiliation of Miguel-Ángel Martínez-González was originally given as Harvard School of Public Health.

The correct affiliation for Miguel-Ángel Martínez-González is the University of Navarra, Carlos III Health Institute and Institute of Health Research of Navarra.

The article has been corrected.
